# High affinity is insufficient for strong B cell activation by HIV broadly neutralising antibodies

**DOI:** 10.1038/s41541-026-01468-y

**Published:** 2026-05-02

**Authors:** Chloe Rees-Spear, Olivia Payne, Emma Touizer, Alan Kennedy, Luke Muir, Peter Thomas, Alyssa Thomas DeCruz, Rachel A. McKendry, Leo Swadling, Marit J. van Gils, James E. Voss, Laura E. McCoy

**Affiliations:** 1 Institute for Immunity and Transplantation, UCL, London, UK; 2 London Centre for Nanotechnology, UCL, London, UK; 3 UMC, Amsterdam, the Netherlands; 4 The Scripps Research Institute, La Jolla, CA USA

**Keywords:** Microbiology, Infection, Immunology, Humoral immunity

## Abstract

HIV broadly neutralizing antibodies (bnAbs) have been widely studied, and inducing a robust broadly neutralizing response remains a goal for many human vaccine studies. Much research focusses on creating higher affinity antigens to target bnAb precursors, however this ignores the role of non-neutralisers that are known to dominate HIV-specific responses. Furthermore, any effective vaccine will likely need to induce multiple bnAbs. Here, we question whether the previously postulated affinity ceiling of 10^-9^M limits bnAb induction above a certain affinity. Using adeno-associated virus (AAV)-mediated CRISPR-Cas9 engineering to express HIV-specific bnAbs and non-bNAbs in Ramos B cells, we investigate antigen-specific activation by BCRs of known binding/neutralization potency, epitope and affinity. We found that between different bnAb epitopes affinity was not predictive of activation. However, within individual epitopes and across non-bnAb epitopes, increased affinity results in increased activation. We propose that at activation-resistant bnAb epitopes, affinity is overruled to an extent by the physical BCR-antigen interaction, while more ‘simple’ and immunogenic non-bnAb epitopes are more heavily dependent on BCR affinity. These findings may have significant consequences for vaccine development, as a high affinity BCR-antigen interaction at an epitope with poor activation potential may be unsuccessful at participating in the humoral response.

## Introduction

To date, HIV-1 has been acquired by an estimated 88.4 million people globally, with 40 million people living with HIV (PLWH) today^1^. The impact of HIV has been somewhat lessened in recent years, thanks to the development of effective antiretroviral therapy (ART)^2^. Despite these advances, importantly, ART is a treatment, not a cure, and the only way to truly prevent new infections is development of an effective HIV vaccine. This has been challenging because mimicking the antibody response to HIV infection does not produce an effective vaccine. This is because the antibody response after natural infection is not effective at clinically controlling viraemia or preventing superinfection, as the antibodies induced are generally neither sufficiently potent nor broad enough to prevent viral escape^3,4^. The HIV-specific humoral response is thus dominated by low-affinity, strain-specific or non-neutralising antibodies (non-bNAbs)^5,6^. Generally, a few years post-acquisition of HIV, 10–30% of individuals may produce antibodies capable of neutralising several autologous strains, and only 1–10% of these PLWH go on to produce bnAbs^3,7,8^. Although bnAbs themselves have been well characterised, the cells that produce them are still a relatively unknown quantity. The cause of bnAb rarity during the course of natural infection is unknown, but is likely due to a combination of factors such as ongoing immune dysregulation and development of auto and/or polyreactivity^9^. In particular, prevalence of low affinity precursors is thought to be a particular hindrance to bnAb induction^10^. Development of a broadly neutralising response is complex, often involving high levels of somatic hypermutation (SHM), some level of autoreactivity and the use of unusually long CDRH3s^11–13^. Entry of bnAb precursor cells into germinal centres (GC), where they acquire high levels of SHM, is made even more difficult by an affinity barrier to GC participation.

The target for many HIV vaccine candidates is the induction of bnAbs, defined as being capable of neutralising more than 7 different clade viruses when tested against a panel of 118 pseudoviruses^14,15^. To date, no vaccine candidate has robustly induced a broadly neutralising response in humans. However, some bnAbs have been induced after vaccination in wildtype animals^16,17^, including a recent study triggering cross-neutralising serum antibody responses in all immunised non-human primates^18^. Several studies have demonstrated that germline reverted bnAbs are unable to bind HIV envelope glycoprotein (Env), requiring modified antigens with higher affinity for germline bnAbs to induce GC responses^19–22^. Many vaccine candidates, in particular those for germline-targeting vaccines^23^, thus aim to expand a small pool of precursors with germline targeting immunogens, then broaden responses with subsequent vaccinations^24,25^. Therefore, the issue of how these rare bnAbs are induced in infection, and how to replicate their development in a vaccine setting, is still an open question.

Despite the development of neutralising antibodies, although not bnAbs, in the majority of PLWH, effective humoral responsiveness is hampered not only by rare bnAb precursors but also by the immune dysregulation that occurs concurrent with untreated infection. Chronic infection results in B-cell hyperactivity and gammaglobulinemia (Schnittman et al.,^26^; Shirai et al.,^27^), impaired responsiveness to antigen^28,29^, and significant alterations in the proportions of memory B cell (MBC) phenotypes in PLWH. Of these, atypical B cells (CD21- CD27-) usually form a small transient population in healthy individuals following infection and vaccination but are expanded in various chronic conditions^30–34^, and have been linked to impaired B cell responsiveness^35,36^. If, in HIV infection, rare neutralising antibody precursor B cells are trapped in this poorly responding atypical population, then this may further contribute to the rarity of bnAbs within an individual.

BnAbs have been extensively studied in the form of monoclonal antibodies (mAbs)^23,37^; however, there is a significant gap in understanding how they behave on the cell surface and what effect that has on bnAb generation. Very few studies have comprehensively investigated downstream cellular effects as a result of bnAb BCR expression outside of transgenic mice studies, or how BCR behaviour relates to characteristics such as affinity of the bnAb in question. Furthermore, non-bNAbs are often overlooked in the search for ever more potent bnAbs despite their known role in modulating bnAb development^5,38–41^.

Here, we first interogate the ability of HIV-specific primary MBC subsets to respond to HIV Env antigen. We then engineered a selection of well characterised, high affinity bnAbs along with a selection of autologous non-bNAbs into Ramos B cells using AAV-mediated CRISPR-Cas9^42^, a common technique for in vitro investigation of specific antibody responses^43,44^. Using these engineered cells, we investigated whether HIV Env-specific B cells followed the immunological dogma that greater BCR affinity results in greater cellular activation^45–48^, or adhered to the concept of an affinity ceiling proposed by Batista and Neuberger, whereby there is an upper limit to activation as a result of affinity^48–50^. Contrary to immunological dogma, we did not observe a simple linear relationship between affinity and activation in bnAbs, as compared to non-bNAbs. We postulate factors such as BCR stoichiometry, antigenic footprint, and angle of approach sufficiently modulate B cell activation such that affinity metrics are overruled above the affinity ceiling of 10^−9^M in determining B cell activation. Thus, particular bnAb epitope targets may be at a disadvantage due to the nature of the BCR-Env interaction, even if they have matured comparable affinity to antibodies against other more favourable epitopes. As a successful HIV vaccine will likely require a multi-epitope response, these findings suggest that different strategies will be required beyond boosting the affinity of vaccine antigens above the affinity ceiling to induce a strong bnAb response against multiple epitopes.

## Results

### Antigen-specific cells are too rare in PLWH to assess their activation by HIV Env

**Fig. 1:**
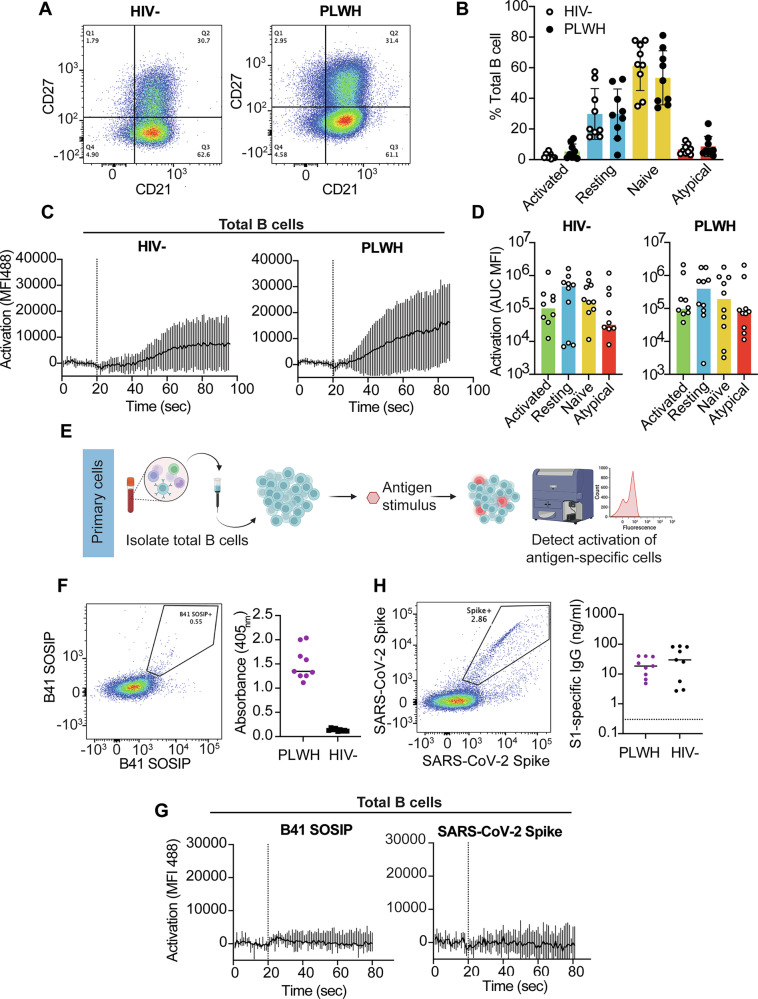
Antigen-specific cells are too rare to assess differences in activation in response to HIV Env. **A** Representative staining of MBC phenotypes from PLWH and HIV- individuals. **B** Percentage of total, CD10-CD19+ B cells in PLWH and HIV- individuals. **C** Average calcium mobilisation in total primary B cells from PLWH (*n* = 9) and HIV- (*n* = 9) individuals, measured as change in AF488-MFI over time following stimulation with anti-kappa/lambda (1 mg/ml), normalised to average baseline. Solid line in calcium mobilisation indicates median activation, with bars indicating 95% confidence interval. Vertical dotted line indicates stimulation time point. **D** Activation of MBC phenotypes in **C** represented as area under the curve (AUC) of AF488-MFI following activation, baseline subtracted. **E** Diagram of antigen specific activation of primary B cells and detection of calcium mobilisation. **F** Representative flow staining (left) and serum binding (right) to B41 SOSIP. **G** Average calcium mobilisation in total primary cells from PLWH following stimulation with HIV antigen B41 SOSIP (left) or with SARS-CoV-2 Spike protein (right) (6 μg/ml), normalised to average baseline. Solid line in calcium mobilisation indicates median activation, with bars indicating 95% confidence interval. Vertical dotted line indicates stimulation time point. **H** Representative flow staining (left) and serum binding (right) to SARS-CoV-2 S1 subunit. Diagram in E created in BioRender. Rees-spear, C. (2025) https://BioRender.com/8qdo7zj.

First, given prior observations of widespread B cell defects during HIV^33,51,52^ we investigated if there was an intrinsic difference between PLWH and HIV negative individuals in the ability of their total B cells to activate in response to BCR stimulation. For this, a group of 17 study participants (PLWH = 9, HIV-negative = 9) were selected for assessment of their circulating B cells. B cells were isolated and stained for memory phenotype according to expression of CD21 and CD27 (Fig. 1A and Supplementary Fig. 1). Among this cohort, there were no significant differences in the proportion of MBC phenotypes between PLWH and HIV-negative individuals (Fig. 1B). This is likely due to the relative normalisation of MBC phenotypes as a result of ART^53–55^. Cells were further stained with fluorescent indicator Fluo-4, which binds free calcium within the cell^56^. To stimulate total primary B cells, antibodies targeting the kappa and lambda light chains were added to crosslink BCRs and activate the cells. There was no significant difference in average total B cell activation (Fig. 1C) or across MBC subsets (Fig. 1D) following anti-kappa/lambda BCR stimulation in PLWH compared to HIV-negative individuals, as expected given the success of their ART treatment in normalising any MBC defects (Fig. 1B). Within the MBC subsets, we observed that resting and naïve MBCs were, on average, the most responsive to BCR stimulation (Fig. 1D), although these differences were not significant due to sample size. Cliff’s delta was applied to quantify the magnitude and direction of calcium flux trends across primary B cell subsets; directional trends were inferred when the 95% confidence intervals did not include 0. Activated and atypical cells displayed a medium trend towards lower calcium flux following stimulation (activated/atypical vs resting/naive = −0.27 (CI −0.49 to −0.02)), with most atypical cells exhibiting minimal activation (Fig. 1D), in agreement with prior studies^32,36,57^. Thus, we have demonstrated that there is no overall defect in the ability of B cells in PLWH on ART to respond to generic BCR stimulation. We therefore investigated the ability of these cells to respond to HIV antigen-specific stimulation.

To investigate the antigen-specific response of primary B cells (Fig. 1E), we screened individuals for high serum binding to B41 SOSIP (a trimer-stabilised, clade B HIV Env), as it was most likely to be recognised by the cohort sampled who have predominantly acquired a clade B strain (Fig. 1F). The top ten individuals in terms of serum reactivity were selected to test for antigen-specific activation. However, when stimulated with B41 SOSIP, there was no detectable B cell activation across all samples as measured by calcium flux (Fig. 1G). We postulated that this was likely due to the low prevalence of HIV-specific B cells within this group (<0.5%, Fig. 1F). To confirm this, we tested stimulation with another antigen, SARS-CoV-2 Spike protein, in a cohort that had been sampled in the autumn/winter period of 2022 and therefore displayed elevated Spike-specific serum and MBCs (Fig. 1H)^31^. Although these individuals had greater Spike binding (Fig. 1H)^31^, measureable activation could not be detected by calcium flux (Fig. 1G). All samples were run with an anti-kappa/lambda stimulus as positive control to ensure technical errors were not the cause of the lack of antigen-specific signal. Thus, antigen-specific primary cells are too rare for activation to be measured by calcium flux even at relatively high frequencies.

### CRISPR engineering of B cell line enables investigation of antigen-specific activation

**Fig. 2:**
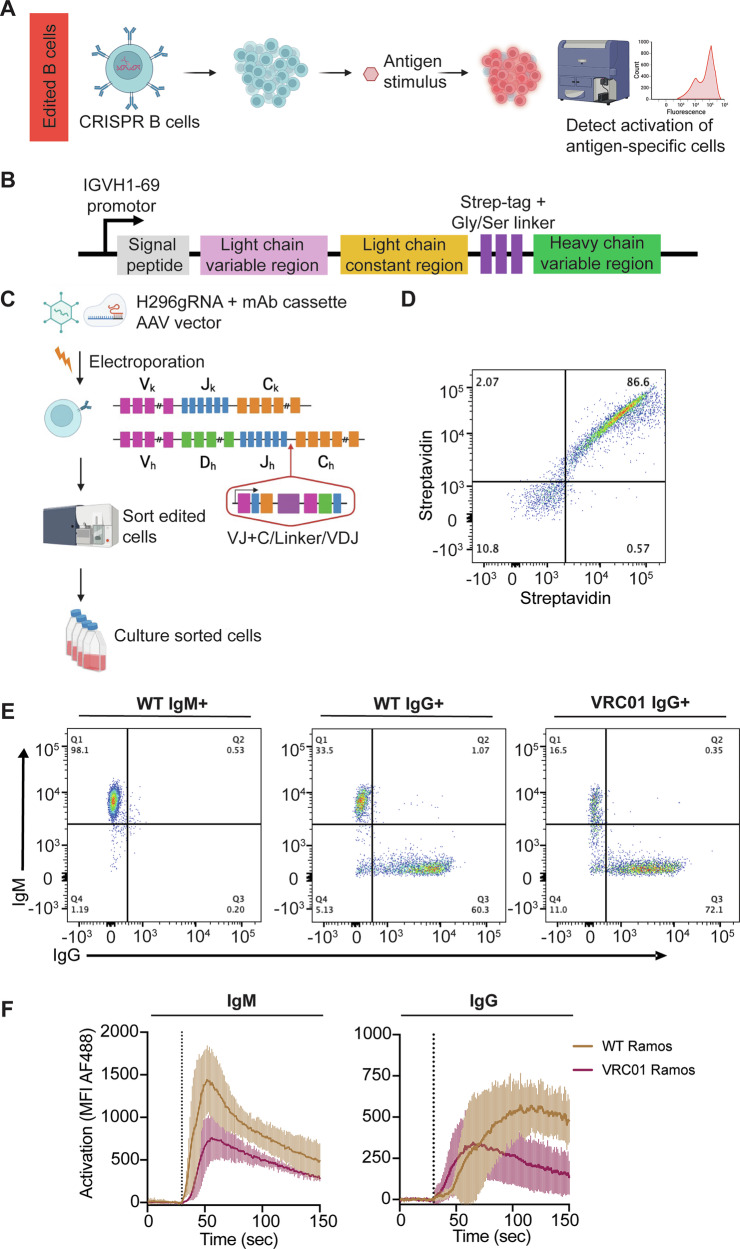
CRISPR-Cas9 engineering of Ramos cells allows expression of functional BCR of interest. **A** Diagram of CRISPR engineering of desired BCR into Ramos B cells, enabling assessment of antigen-specific activation by calcium flux by increasing the number of responding cells. **B** Diagram of the antibody expression cassette within AAV carrying complete light chain, and heavy chain VDJ, under control of the IGVH1-69 promoter. A glycine-serine linker prevents recombination with the endogenous light chain, and a Strep-tag enables detection of edited cells. **C** Diagram of the CRISPR engineering method whereby Ramos B cells are electroporated with pre-complexed RNPs targeting the endogenous heavy chain and infected with AAV carrying the BCR of interest. Cells are then sorted based on expression of the inserted Strep-tag. **D** Representative flow plot showing that AAV-mediated CRISPR-Cas9 results in high efficiency editing of the Ramos BCR. **E** Representative flow plots of Ramos cells expressing unedited WT (left) or CRISPR-induced class-switched unedited WT (middle) and class-switched bnAb VRC01 (right) BCRs. Cells were subsequently sorted to create pure IgM+ and IgG+ populations. **F** Representative plots showing calcium flux of IgM (left) and IgG (right) WT and VRC01-expressing Ramos cells after stimulation with anti-kappa/lambda antibody, demonstrating functionality of the inserted BCR. Diagrams in A and C created in BioRender. Rees-spear, C. (2025) https://BioRender.com/8qdo7zj and https://BioRender.com/c3zcvsw, respectively.

As the proportion of primary B cells specific for the target antigen is too small to measure any alterations in activation in either chronic HIV or recently vaccinated individuals in this system, we used CRISPR-Cas9 engineering of the Ramos B cell line to create a line of B cells expressing the antibody of interest from the immunoglobulin locus in a controlled setting. Thus, this system would create a larger population of known B cells with which to assess activation following specific stimulation (Fig. 2A). BnAb-expressing Ramos B cell lines were generated using an AAV-mediated CRISPR-Cas9 system to enable expression of the antibody at the endogenous Ig locus as previously described^42^ (Fig. 2B, C). Expression of a Strep-tag within the antibody cassette enabled confirmation of >40% editing efficiency (Supplementary Fig. 2A) that was increased to >80% positive expression of the engineered bnAb across each cell line after sorting (Fig. 2D and Supplementary Fig. 2D). A representative flow plot of cells expressing VRC01+Strep-Tag is shown in Fig. 2D. The Ramos B cell line expresses IgM as the surface BCR^58^ and, as the CRISPR editing strategy does not alter the heavy chain constant region, all edited cell lines were initially IgM+. To fully assess activation as a result of mature bnAb BCRs, CRISPR was once again employed to achieve class switched cells, by inducing dsDNA breaks before the IgM locus and before the IgG1 locus (Supplementary Fig. 2B, C)^59^. This triggers natural class switch recombination by the cell, resulting in the BCR switching to IgG1. Unedited (WT) Ramos cells (Fig. 2E, left) were class switched to IgG1 (Fig. 2E, middle). A representative edited bnAb cell line expressing VCR01 IgM was similarly class switched to IgG1 (Fig. 2E, right). Cells were subsequently sorted for IgG expression to achieve pure IgM+ or IgG+ cell lines. Stimulation of the resulting edited IgM or IgG BCR results in measureable calcium mobilisation when stimulated with anti-kappa/lambda antibody (Fig. 2F). Activation of bnAb engineered cells is reduced compared to WT, likely as a result of reduced BCR expression from the AAV mAb cassette compared to the WT locus, as observed elsewhere^60–62^ (Fig. 2F and Supplementary Fig. 2D). However, this technique provides a tool with which to measure specific responsiveness to antigen for B cells expressing known mAbs on the surface of the cell.

### Cellular activation is determined by more than affinity or neutralisation potency in bnAb expressing B cells

To further understand how antibody affinity of HIV bnAbs might relate to activation of their parent B cell following stimulation, four different bnAbs were selected to cover the major bnAb epitopes on Env: CD4bs (VRC01^11^), high mannose patch (PGT121^63^), apex (PG9^64^) and the gp120-gp41 interface (ACS202^65^). Together, these bnAbs form a representative group that binds the major HIV neutralising epitopes and neutralise Tier 2 viral strains, making them ideal for testing how the affinity of potent HIV bnAbs is related to their ability to activate their parent cell in response to Env. BG505 was used here as there is no autologous antigen for many of these bnAbs and BG505 is the most widely used HIV Env SOSIP antigen^66–71^. AMC011 was chosen because it represents the autologous virus for bnAb ACS202, providing a unique opportunity to investigate the bnAb response to its native virus. After production of the bnAbs in HEK293F expi cells, we validated each antibody’s neutralisation function and binding affinity by Tzm-bl pseudovirus neutralisation assay, ELISA and biolayer interferometry (BLI), respectively (Supplementary Fig. 3A–C).

**Fig. 3:**
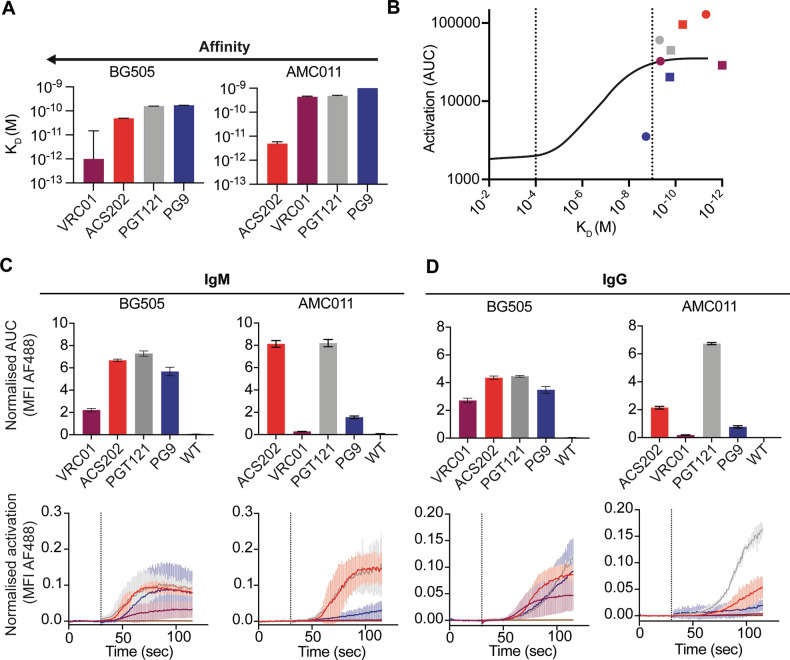
Neither affinity nor neutralisation potency determine cellular activation in bnAb expressing B cells. **A** Affinity of bnAbs against BG505 (left) and AMC011 (right) as measured by BLI, in order of decreasing affinity (left to right) against each antigen. Error bars indicate standard error. WT indicates unedited Ramos B cells. **B** Diagram bnAb affinity and activation by AMC011 (dots) and BG505 (squares) SOSIPs in relation to the affinity ceiling. Theoretical affinity-activation relationship shown in black. Dashed lines indicate affinity threshold (lower) and ceiling (upper) to activation. ACS202 (red), PGT121 (grey), VRC01 (maroon), PG9 (blue), WT (brown). Calcium flux of indicated bnAbs when expressed as IgM (**C**) or IgG (**D**) following stimulation with BG505 (left) and AMC011 (right) SOSIPs. Area under the curve (AUC, top) is calculated from time-course plots (bottom) using PRISM software. Error bars indicate standard deviation across three experimental repeats. See Supplementary Table 1 for statistical analysis.

As expected, all bnAbs tested demonstrate nanomolar affinity for both Env antigens, with affinity constants (*K*_D_) ranging between 10^−9^ M and 10^−12^ M for AMC011, and 10^−10^ M and 10^−12^ M for BG505 (Fig. 3A and Supplementary Fig. 3C, E). ACS202 demonstrated the highest affinity for AMC011, dropping a log-fold against BG505 SOSIP (10^−12^–10^−11^M). VRC01 has the highest affinity for BG505 SOSIP, dropping almost 2 logs against AMC011 SOSIP (10^−12^–10^−10^M). PGT121 demonstrates similar affinity for both antigens (within 10^−10 ^M range). PG9 has the lowest affinity for AMC011 SOSIP of all antibodies tested (10^−9^ M), and intermediate affinity for BG505 SOSIP (10^−10^M). Complementing assessment of published bnAb affinities and breadth (Supplementary Fig. 3B), none of the bnAb binding kinetics measured here correlated with neutralisation potency (Supplementary Fig. 3D). It is notable that almost all the affinities measured here are greater than the postulated affinity ceiling of 10^−9^M^45,48^ (Fig. 3B). Although, these observed affinities are higher than some reported affinity values for these bnAbs^65,67,72^, which may be due to differences in affinity measuring techniques^73,74^.

To assess the relationship between affinity and activation, release of intracellular calcium was measured by flow cytometry for 90 seconds following antigen stimulation of the Ramos bnAb cells. Subsequent Fluo-4 MFI readings were normalised to the BCR MFI to account for differential BCR expression across engineered cell lines (Supplementary Fig. 2D), although this does not significantly alter overall activation patterns across cell lines (Supplementary Fig. 2E). Area under the curve post-stimulus was calculated for each time course to compare overall activation. Cells were stimulated with either BG505 or AMC011 Env, as IgM and IgG BCR (Fig. 3C, D). For bnAb IgM+ cells (Fig. 3C), activation of the cells showed no correlation with overall affinity, and notably all affinities for BG505 SOSIP were greater than the affinity ceiling (10^−9^M). Activation by BG505 resulted in highly variable activation between antibodies, with statistically different calcium flux between almost every bnAb BCR (Welch’s *t*-test, Supplementary Table 1). VRC01 displays the highest affinity for BG505 SOSIP (1 × 10^−12^M, which is the upper limit for BLI measurements, Fig. 3A), yet activation of cells expressing this bnAb is lower than that seen with other antibodies with lower affinity such as ACS202 (which has half a log-fold affinity lower at 4.9 × 10^−11^M) (Fig. 3C). Surprisingly, lowest affinity bnAb PG9 results in greater calcium flux following BG505 SOSIP stimulation than highest affinity VRC01 (Fig. 3A, C). For AMC011 SOSIP, activation of IgM+ ACS202 and PGT121 B cells results in the greatest level of activation. However, there is minimal activation of VRC01 or PG9 cell lines upon stimulation with AMC011. This does not appear to be due to a lack of affinity, as PGT121 has similar affinity for AMC011 as VRC01 (Fig. 3A, C). Once again, there does not appear to be a linear relationship between affinity of these bnAbs for SOSIP and the level of activation their parent cell receives.

For IgG+ cells, the pattern of activation matches that of the IgM+ cells when stimulated with both BG505 and AMC011 SOSIPs (Fig. 3D). This was predominantly as expected, given the VDJ regions of these cell lines are not altered by the induced class-switch recombination^75^. Following stimulation with BG505 SOSIP, activation of IgG+ cells is slightly slower for all bnAbs compared to the IgM+ cells (as shown in the kinetic curves in Fig. 3C, D) but reaches a slightly higher peak. When stimulated with AMC011 SOSIP, ACS202 and PGT121 are the only two IgG cell lines that respond robustly to stimulation (Fig. 3D).

These results demonstrate that across different bnAbs, BCR affinity is not predictive of B cell activation, and that some other aspect of this antigen-BCR interaction is modulating the resulting activation the parent B cell receives.

### Within individual bnAb epitopes, changes in affinity determine changes in cellular activation

**Fig. 4:**
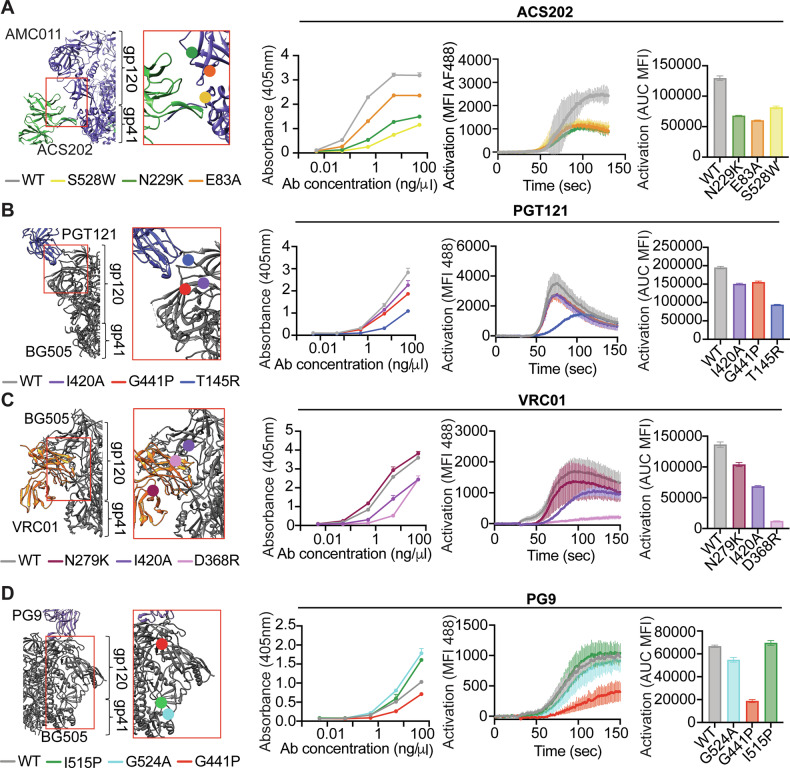
Within individual bnAb epitopes, changes in affinity determine the level of cellular activation. Structural diagrams (left) of bnAbs ACS202 (**A**), PGT121 (**B**), VRC01 (**C**) and PG9 (**D**) bound to AMC011 (purple) or BG505 (grey) SOSIPs. Red box indicates magnified region. Coloured dots indicate sites of point mutations. Binding of bnAbs to mutated SOSIPs was tested by ELISA (middle left) and BLI (Supplementary Fig. 4). Cells expressing each bnAb were assessed for calcium flux (middle right: time course; right: AUC normalised to baseline) following stimulation with the indicated mutant SOSIP. WT indicates unedited Ramos B cells. PDB: 6NC2, 7UOJ, 6V8X, 7T77.

As antibody affinity did not appear to be a determinant of B cell activation in these bnAb cell lines, we questioned whether bnAb stoichiometry may play a role in moderating the observed calcium flux, given the bnAbs target different epitopes, with some binding only once per trimer (PG9) and some up to three times (ACS202, VRC01). Stoichiometry of binding may have significant impacts on the ability of a bnAb to activate the cell, as greater binding stoichiometry may result in greater cross linking of the BCR^76^, thus contributing to the lack of a clear relationship between bnAb affinity and antigen specific activation. To address this question, point mutations were introduced into the AMC011 and BG505 SOSIP antigens to specifically reduce binding affinity of each bnAb BCR (Fig. 4 and Supplementary Fig. 4). Investigation of low versus high affinity antigen-BCR interactions at the exact same epitope could thus be assessed in terms of activation without the complication of the BCRs targeting different epitopes at different stoichiometries.

Within each epitope, changes in affinity do result in relative changes in activation. For ACS202, when stimulated with lower affinity AMC011 SOSIP mutants, activation was nearly halved for all mutants compared to WT AMC011 stimulation (Fig. 4A). All mutations in AMC011 resulted in a 10^−10^M affinity (compared to 10^−11^M for WT) and produced roughly equal reductions in activation. Similarly, for PGT121, BG505 T145R resulted in the biggest drop in affinity (10^−9^M) along with the lowest level of activation (Fig. 4B). BG505 G441P and I420A, both resulted in less than a log reduction in affinity compared to WT, resulted in only a small decrease in activation corresponding to their small drop in affinity. Average activation of VRC01 by mutant N279K was lower than WT, matching its lower affinity (Fig. 4C). Mutant I420A demonstrated an intermediate knockdown in affinity, resulting in an intermediate level of activation. Stimulation with mutant D368R resulted in almost no activation of VRC01 B cells, which is not surprising given its nearly 3-log drop in affinity (Supplementary Fig. 4). Activation of PG9 was weak overall, which was not unexpected given its 1:1 binding stoichiometry (Fig. 4D). WT, I1515P and G524A all resulted in similar levels of activation of PG9 despite roughly a log-fold difference in affinity. In particular, I515P has the lowest measured affinity (although only marginally compared to the other mutants) yet induced the highest level of activation. Only G441P resulted in a notable decrease in activation compared to WT. It should be noted that, unlike the other bnAb epitopes, the mutations used for PG9 do not sit directly within the apex epitope as mutations within this site tend to ablate binding completely (Fig. 4D). Thus, while mutations for the other bnAbs weaken contact points within the epitope, G524A, G441P and I515P likely result in conformational changes at the trimer apex to alter how PG9 binds, as these mutations are distant from the apex epitope.

Overall, the data presented here indicate that within an individual epitope-paratope interaction, affinity directly impacts the level of activation the cell receives. Thus, the dogma of increased affinity resulting in increased activation holds true for complex antigens but only within certain contexts. Activation clearly increases in line with increased affinity greater than the theorised 10^-9^M within specified interaction sites, where affinity determines the level of activation. Furthermore, these data suggest that a defined ceiling to affinity maturation may be true for development of a single antibody lineage, but may be much more flexible in the development of a polyclonal antibody response against a more complex antigen. While we only present a small number of antibodies here, B cell activation within an epitope appears to be able to increase with greater affinity up to at least 10^−12^M.

### Affinity dependent activation of non-bNAb BCRs

To fully understand how antibody affinity influences B cell fate in the setting of a highly variable, chronic infection such as HIV, we considered the role of the other Env-specific antibodies that exist within an ongoing immune response in addition to bnAbs. Autologous neutralising antibodies in those with bnAb activity have been suggested to play a role in bnAb development^40^, but limited data have been produced investigating the relative activation of their parent cell. We hypothesised that B cells expressing strain-specific or non-neutralising antibodies, which bind antigen with affinities at least equal to that of bnAbs, may receive the same level of positive selection via B cell activation, but this has yet to be thoroughly investigated. To test this, we selected 7 groups of somatic variants isolated from the ACS202 bnAb donor for which the autologous virus (AMC011) was also available^65,77,78^.

**Fig. 5:**
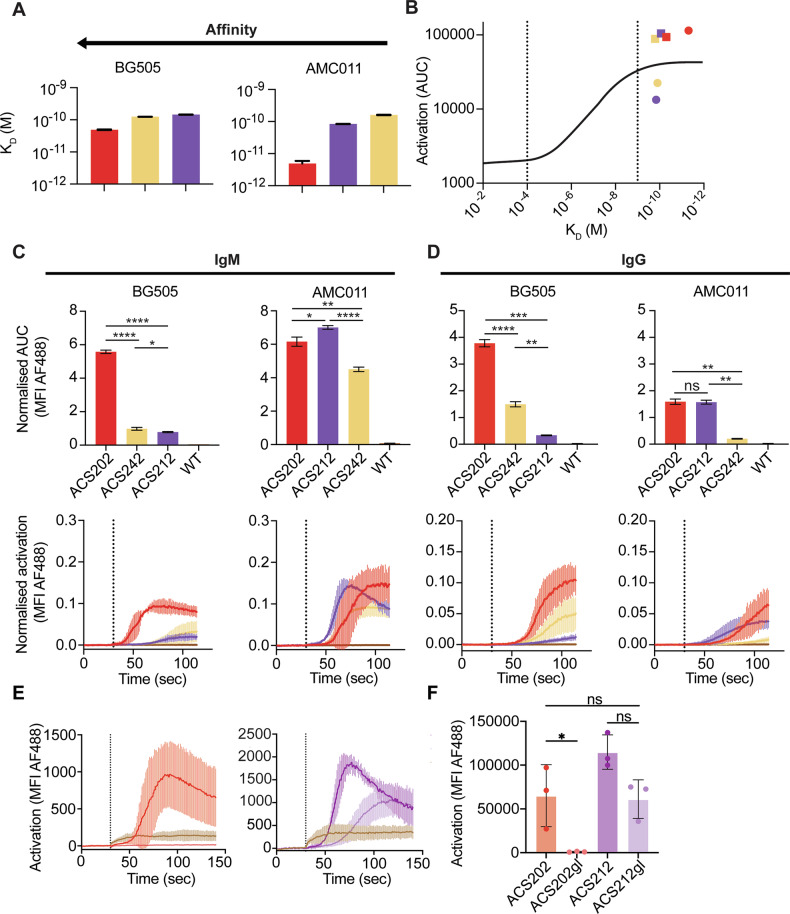
ACS autologous non-NAb affinity dictates cellular activation. **A** Affinity (*K*_D_) of ACS bnAb ACS202 and autologous non-bNAbs ACS212 and ACS242 for BG505 (left) and AMC011 (right) SOSIPs. Arranged in order of decreasing affinity left to right. WT indicates unedited Ramos B cells. **B** Diagram of ACS antibody affinity and activation against AMC011 (dots) and BG505 (squares) SOSIPs in relation to the affinity ceiling. Theoretical affinity-activation relationship shown in black. Dashed lines indicate affinity threshold (lower) and ceiling (upper) to activation. ACS202, red; ACS212, purple; ACS242, yellow. Area under the curve (AUC, top) and time course (bottom) of change in MFI-AF488 following stimulation with BG505 (left) or AMC011 (right) in IgM+ (**C**) and IgG+ (**D**) cells. ****P < 0.0001, ***P < 0.0005, **P < 0.005, *P < 0.05, Welch’s *t*-test. **E** Calcium flux of IgM+ B cells expressing mature or inferred germline versions of bnAb ACS202 (left) or non-bNAb ACS212 (right) stimulated with AMC011 SOSIP. *n* = 3. **F** AUC of activation in **F**. *P = 0.0375, ns = *P* > 0.05 (one-way ANOVA).

Validation of binding and neutralisation potencies demonstrated that antibodies from each of the clonal families bind AMC011 and BG505 Env antigens with a range of binding strengths and demonstrate weak to no neutralisation of an autologous virus and no neutralisation breadth against Tier 2 viruses (Supplementary Fig. 5A) and so are termed non-bNAbs. Despite poor neutralisation capacity, BLI assessment of affinities demonstrated higher than nanomolar affinity for all non-bNAbs tested (Supplementary Fig. 5B). Three representative antibodies targeting different epitopes and with variable neutralisation function were then selected for Ramos CRISPR engineering: the bnAb ACS202, along with two of the highest affinity non-bNAbs, ACS212 (targeting the V3 loop) and ACS242 (targeting the CD4bs) (Fig. 5A, B and Supplementary Fig. 5C).

All IgM cell lines showed activation upon stimulation by both AMC011 and BG505 SOSIPs (Fig. 5C, D). Interestingly, contrary to the pattern seen with the bnAbs from different donors, the autologous antibodies do tend to show a decrease in activation in line with decreasing affinity. There is a clear pattern of statistically significant decreases in activation with each step down in affinity. In IgM+ cells, bnAb ACS202 responds potently to BG505 stimulation in line with its having the highest affinity (Fig. 5C). ACS242 and ACS212 have similar affinities and both respond poorly to BG505 stimulation relative to ASC202, with the higher affinity ACS242 making a slightly better response (Fig. 5C). Against the autologous AMC011 SOSIP, both non-bNAb B cells are well activated in line with their greater affinity for this antigen. ACS212 achieves stronger overall calcium flux than bnAb ACS202, but the kinetics of the activation are shorter, with calcium flux peaking sooner than ACS202 but then dropping off rapidly. Following the pattern of affinity across these three antibodies, the lowest affinity antibody ACS242, is also the poorest responder to AMC011 stimulation.

When class switched to IgG, the pattern of activation in these cell lines is similar to their IgM+ counterparts, as expected given their VDJ region remains the same (Fig. 5D). Against AMC011 SOSIP, there is no significant difference in the level of activation between bnAb ACS202 and non-bNAb ACS212. When stimulated with BG505 SOSIP, ACS202 IgG responds most potently, although slightly weaker than in IgM+ cells. ACS242 IgG+ cells respond poorly when stimulated with BG505 SOSIP and demonstrate very little activation following AMC011 SOSIP stimulation. In a similar pattern to its IgM+ cell line, ACS212 shows almost no activation by BG505 SOSIP, matching its low affinity. These results suggest that although epitope-specific interactions may significantly alter bnAb BCR activation when comparing bnAbs from different individuals, this influence is less apparent when considering a small group of neutralising and non-neutralising antibodies from one individual who made a broadly neutralising response. However, investigation of a larger group of non-bNAb BCRs is required to determine if these results are a feature of all non-bNAbs.

### Inferred germline ACS bnAb and non-bNAb result in similar levels of activation

Thus far, our investigation of HIV-specific B cell activation suggests that although bnAbs are high affinity, this does not guarantee stronger activation of their parent cell than other cells expressing bnAb BCRs with relatively lower affinity for antigen. Instead, it appears that at high affinities, bnAb activation may be modulated by the nature of epitope-specific interactions and that if non-bNAbs can achieve similarly high affinity they may activate to a similar extent. Thus, a scenario is plausible that within an ongoing HIV-specific response, a B cell expressing an autologous non-bNAb that achieves sufficiently high affinity (>10^−9^M) may achieve more positive selection than a bnAb-expressing cell that targets an epitope with inefficient activation potential, regardless of the bnAb having extremely high affinity. Notably, all but one of the non-bNAbs studied here achieved this high affinity interaction. However, upon first encountering antigen, the naïve B cells that evolve to produce either type of antibody likely have a different binding capacity to their mature forms as they are yet to affinity mature against the target antigen. It is well known that most bnAbs fail to bind Env in their germline conformation^22,79–81^. However, there is some evidence that non-bNAbs, being less highly mutated, retain some ability to bind Env in their germline form, while bnAb precursors require specifically tailored antigen^19^. Thus, it is likely bnAbs are at an additional disadvantage on initial antigen encounter.

To test this hypothesis here, selected ACS antibodies were reverted to their inferred germline sequence as previously described^21,79,81^ for bnAb ACS202 and its autologous non-bNAb ACS212. Each germline was tested for binding and neutralisation of autologous AMC011 (Supplementary Fig. 5D, E). As expected, the bnAb ACS202gl showed no detectable binding or neutralisation. Meanwhile ACS212gl was still capable of binding AMC011, with only a slight reduction compared to its mature form, but remained unable to neutralise (Supplementary Fig. 5E). As expected, when assessed by BLI, the inferred germline for bnAb ACS202 demonstrated extremely low affinity against autologous AMC011 SOSIP with a K_D_ below the accurate limit of detection on the BLI instrument (1 × 10^−5^M) (Supplementary Fig. 5D). This affinity is also below the threshold for cellular activation previously found for HEL antigen and naïve VRC01-class B cells (1 × 10^−6^M)^10,48,82^. Non-bNAb ACS212 on the other hand, when reverted to germline has a nanomolar affinity of 1.18 × 10^−9^M, therefore demonstrating affinity above the ceiling of activation before any maturation has occurred (Supplementary Fig. 5D). As expected, when stimulated with AMC011 Env, B cells expressing ACS202gl fail to activate (Fig. 5E). ACS212gl on the other hand retains strong activation, although with a slightly lower and delayed peak activation compared to the mature BCR (Fig. 5E). Comparing total activation, ACS212gl activates much more in response to AMC011 SOSIP compared to ACS202gl (Fig. 5F). Notably, there is no significant difference in activation between mature ACS202 and ACS212gl (Fig. 5F). These data suggest that, within the same host, when reverted to their inferred germline, non-bNAbs may be better able to bind Env and result in significantly greater cellular activation than a bnAb precursor, even showing similar levels of activation to a mature bnAb. Therefore, a contributing factor to the dominance of poorly neutralising antibodies compared to a bnAb from the same donor may be the result of a ‘head-start’ phenomenon within the GC for non-bNAbs, as well as lack of affinity differential between non-bNAbs and bnAbs after maturation. However, comparison across a greater number of autologous non-bNAbs is needed to draw strong conclusions as to how these germline B cells may interact, and the presence of alternative autologous viral strains presents a further confounding factor in determining how these competing B cells may respond to ongoing infection.

### Antigen acquisition is determined by activation rather than affinity

Although calcium flux signals the initial stages of BCR engagement it does not necessarily indicate successful B cell activation^83^. Successful B cell activation results in the cell receiving survival and proliferation signals in order to progress through the next stage of B cell development^84–86^. To do this, the B cell must acquire antigen from the presenting cell and internalise and process it for presentation to T cells. Affinity has previously been linked to antigen acquisition, whereby the higher affinity the BCR, the more antigen the B cell acquires^87–90^. To measure antigen acquisition, antigen internalisation was detected with the use of streptavidin pHrodo-Red. This is a fluorescent pH indicator conjugated to a streptavidin molecule, which fluoresces more strongly as antigen progresses along the increasingly acidic endocytosis pathway (Supplementary Fig. 6A).

**Fig. 6:**
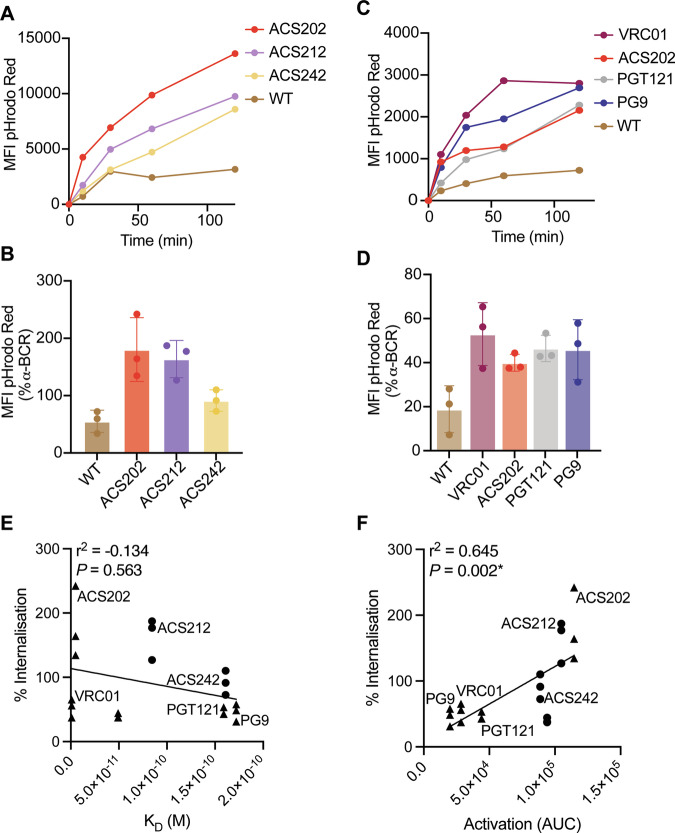
Antigen internalisation correlates with activation rather than affinity. **A** Representative time course of AMC011 SOSIP by cells expressing ACS antibodies. **B** Maximum internalisation of AMC011 SOSIP antigen at 2 h by ACS antibody B cells, shown as a percentage of maximum stimulation by positive control anti-IgM antibody. **C** Representative time course of BG505 SOSIP internalisation by B cells expressing bnAbs. **D** Maximum internalisation of BG505 SOSIP antigen at 2 h by bnAb B cells. WT indicates unedited Ramos B cells. *n* = 3. **E**, **F** Linear regression analysis of percent internalisation versus affinity (*K*_D_, **E**) and activation (AUC calcium flux, **F**) for bnAbs (triangles) and non-nAbs (circles) against AMC011 and BG505 SOSIPs.

The ability of bnAb ACS202 and non-bNAbs from the same individual to acquire and internalise antigen was assessed using autologous AMC011 Env antigen (Fig. 6). A representative time course of antigen internalisation is shown, where bnAb ACS202 rapidly begins internalisation and acquires the most antigen (Fig. 6A). The non-bNAb antibody ACS212 internalises slightly less antigen than ACS202, achieving a lower maximum fluorescence at the 2 h time point (Fig. 6A). The non-bNAb ACS242 internalises the least antigen and is slowest to start, with internalisation at 10 and 30 min equal to that of background WT cells (Fig. 6A). Maximum internalisation (normalised to maximum internalisation by anti-IgM stimulation) after 2 h indicates that bnAb ACS202 acquires the most antigen, followed by ACS212. ACS242 internalises minimal antigen, barely surpassing background WT internalisation levels (Fig. 6B). Thus, the amount of internalisation mirrors the amount of B cell activation and also the affinities across these three antibodies from the same HIV exposure.

BnAb-expressing Ramos cells were similarly assessed for their ability to internalise BG505 SOSIP (Fig. 6C). In the representative time course, VRC01 expressing cells internalise antigen most rapidly and achieve a high level of antigen acquisition, only matched in this instance by PG9 although the kinetics of antigen acquisition are slower in PG9-expressing cells (Fig. 6C). ACS202 and PGT121 internalise equivalent levels of antigen and at the same rate (Fig. 6C). At the final 2 h time point, there is no significant difference in the amount of internalisation between cell lines (Fig. 6D). Interestingly, in bnAb and non-bNAb-expressing cells, antigen internalisation seems to mirror the results of the calcium flux. Whereby across different bnAbs there is no relationship between affinity and downstream cellular processes, while across autologous non-bNAbs affinity does appear to determine cellular activation. In fact, percent internalisation did not correlate with affinity across the antibodies tested here (Fig. 6E), while the level of calcium flux correlated significantly with the subsequent level of internalisation (Fig. 6F). Thus, the internalisation data presented here further demonstrates that the ability of an antigen to activate the cell is not guaranteed by increasing affinity beyond 10^-9^M.

## Discussion

We set out to investigate how BCR affinity impacts B cell activation and subsequent downstream cellular processes within the context of HIV infection, with the aim of elucidating the role of affinity and B cell activation in bnAb rarity within the HIV-specific antibody response in PLWH. In primary B cells, we found no difference in the ability of peripheral B cells in PLWH to respond to BCR stimulation compared to HIV-negative individuals, showing there is no inherent B cell activation defect due to chronic, ART-treated infection. When subdivided into MBC populations, mean activation of activated and atypical MBC subsets in both groups is reduced, consistent with published literature describing these populations as being refractory to stimulation with soluble antigen^51^, however our sample size here is too small to detect any significant differences. Therefore, to investigate the link between affinity of Env-specific BCRs and activation of their parent cells, we used CRISPR-Cas9 AAV-mediated engineering to produce Ramos B cell lines expressing both bnAb and non-bNAb BCRs. Canonically, there is an affinity ceiling to the link between affinity and cellular activation set at roughly 10^-9^M for simple antigen^45,46,48–50^. At affinities greater than this ceiling, activation is expected to plateau as the half-life of the BCR-antigen complex exceeds the rate of internalisation, and thus no further affinity advantage is given to the cell^45,48^. The affinities of the bnAbs tested here were all above this postulated affinity ceiling, and thus we expected to find similar levels of activation following stimulation, as this BCR-antigen half-life should be exceeded. However, stimulation with either BG505 or AMC011 SOSIP revealed that activation of these bnAb B cells does not appear to follow the expected relationship to affinity, and there was no plateau in activation above 10^−9^M, nor was there a continuation of the expected linear relationship between affinity and activation. We propose that differential binding as a result of stoichiometric differences that alter BCR-antigen interactions, alternative angles of approach, or variations in BCR clustering, may explain this observed disconnect between bnAb affinity and activation across different bnAb BCRs.

In our analysis, calcium flux responses were normalised to BCR expression, as we had observed lower activation with antigen agnostic BCR stimulation in our engineered cells compared to WT (Fig. 2) and attributed it to the decreased BCR expression observed in our engineered cells. All engineered BCRs in our model are under the control of the same VH1-69 promotor included in the AAV cassette^42^, thus transcription is expected to be the same. However, it is possible any expression differences may occur post-transcription or translation, a normal process in antibody expression^91^. To our knowledge, a direct link between BCR expression level and activation has not been investigated, though links have been found between BCR expression and isotype and B cell development^92,93^. The question is complicated by the fact that the mechanistic details of B cell activation are still debated^94^. Other work has demonstrated variability in BCR expression in engineered cells^95^ and primary cells^76^, although the method of quantification makes comparison difficult. In their engineered cell lines, He et al. observe no link between BCR expression level and cell activation^95^. We similarly observe no alteration in the overall pattern of activation after normalisation (Supplementary Fig. 2E). However, more detailed knowledge of BCR activation is needed to unpick the requirements of receptor expression levels for activation.

Our next step was to assess if affinity was linearly related to activation when controlling for all different factors of antigen-BCR interactions by considering affinity changes within the same paratope-epitope interaction. Subsequent calcium flux assays demonstrated that, indeed, within an epitope, the expected relationship between affinity and activation holds true, whereby increased affinity results in increased activation, although no plateau in activation was observed. Thus, we concluded that increasing affinity for a particular epitope-paratope pair may result in superior activation when considered in isolation, even beyond the affinity ceiling. However, when considering bnAbs that target different epitopes, having greater affinity for the antigen above the affinity ceiling of 10^−9^M does not mean B cell activation plateaus, nor does it guarantee superior B cell activation with increasing affinity. We propose that what determines optimal responsiveness of a bnAb B cell is more likely a combination of binding stoichiometry and BCR clustering than affinity alone due to the complex nature of bnAb epitopes. This is consistent with previous observations using model antigens, indicating that physical interactions between BCR and antigen (the antigen ‘footprint’) act to modulate the resulting B cell activation beyond the simple affinity of the interaction^76^. Furthermore, the role of antigen spacing in directing BCR engagement and signaling has been well established^96^. Thus, given the different angles of approach required for binding at different bnAb epitopes, spacing between Env antigens likely enhances or inhibits BCR cross linking, and thus activation, regardless of affinity above the 10^−9^M ceiling.

It is also possible that differences in bnAb BCR avidity may further impact BCR activation. Avidity interactions can modulate affinity measurements, whereby one BCR may bind at multiple points to its target trimeric antigen generating a similar effect to elevated affinity. As whole immunoglobulin was used for BLI measurements here it is possible that the observed affinities are skewed by avidity effects, although the experimental set up was modified to minimize bivalent interactions. By reducing sensor loading of immunoglobulin to one-fifth maximum load, the distance between each bound antibody was likely greater than the inter-epitope distance on Env. In vitro, it is possible these bnAb BCRs may have bivalent interactions with tetramerized SOSIP. In fact bivalent interactions have been hypothesized to be a requirement for successful B cell activation by multivalent antigen depending on antigen spacing^96–98^. However, investigation of multimerised germline-targeting Env antigens has been shown to present a complex picture for the role of avidity in activation^75^, and the HIV virion itself likely evades avidity effects due to the low density of Env^99^. Thus, avidity may further complicate the affinity-activation relationship above the affinity ceiling, but further work is needed to unpick its role in modifying bnAb BCR activation. Intriguingly, recent work has suggested that increasing avidity via presentation of antigen on liposomes can robustly elicit cross-neutralising antibody responses in non-human primates^18^, suggesting leveraging avidity may be key to inducing effective vaccine responses.

In addition to considering bnAb expressing cells, we investigated concurrent non-bNAbs as these dominate the Env antibody response and may help or hinder bnAb development^40,100–102^. We found most strikingly that non-bNAbs from a bnAb donor displayed strong abilities to activate their parent B cell in response to antigen stimulation. Furthermore, one non-bNAb BCR retained this ability to bind and stimulate activation to a degree even when reverted to its germline state, while the bnAb from the same individual lost all ability to bind or activate when similarly reverted. Interestingly, in contrast to bnAbs targeting different epitopes from different donors, the non-bNAbs from one bnAb donor tested here do appear to activate their parent cell in a more simple affinity-dependent manner, with weaker calcium flux in response to lower affinity stimulation, but this may be due to the small number of antibodies tested from this individual. However, non-bNAbs are frequently less mutated^19^, target immunogenic and easily accessible epitopes, and are likely less complex in terms of their interaction with antigen. As a result, it would be logical if there were a simpler affinity dependence in their activation pattern, as they more easily interact with their target antigen. Conversely, unusual bnAb characteristics, such as long CDRH3 regions, can impact their physical presence to alter angle of approach and how the BCR interacts with its target antigen. Epitope accessibility, such as the recessed CD4bs or heavily glycosylated apex and high mannose patch epitopes, as well as the requirement for trimeric Env, may also impact how easily a bnAb BCR can bind, and may place limits on how the BCR-antigen interaction occurs. This theory is supported by the correlation observed between affinity and activation compared to the relation between activation and BCR internalisation (Fig. 6E, F). It is notable that, with the exception of ACS202, the non-bNAb-expressing B cells exhibit greater activation and BCR-Env internalisation compared to all bnAbs tested. Therefore, in the small set of antibodies studied here, when non-bNAbs are assessed for calcium flux following stimulation, affinity appears to be the main driver of activation, with greater affinity resulting in greater calcium flux regardless of target epitope. However, when assessing bnAbs targeting different epitopes, affinity and activation do not appear to be linked above the affinity ceiling of 10^−9^M, but nor does activation plateau as previously suggested. Meanwhile, within an epitope, where the physical BCR-antigen interaction remains the same and only affinity is altered, the relationship between affinity and activation is restored, although there continues to be enhanced activation above the previously postulated affinity ceiling. This may be explained by the kinetic segregation model of BCR activation, in which exclusion of inhibitory phosphatases from the immune synapse is what triggers downstream signaling^76,94^. In such a model, a BCR that targets the gp41-gp120 interface epitope (such as ACS202) might result in better exclusion of inhibitory molecules from the BCR cluster compared to an apex antibody (such as PG9) that binds the top of the Env trimer. Indeed, Guenaga et al. demonstrate that a PG9-like BCR activates significantly more when stimulated by a multivalent liposome array compared to soluble antigen as tested here, further supporting the ‘antigen footprint’ model^18^. A larger study comparing multiple bnAbs and non-bNAbs across each epitope would be required to further elucidate the effects of these interactions.

These results add complexity to the concept of the affinity ceiling^45,48^, whereby interactions involving complex antigens must take more factors than simple affinity into consideration, and individual epitopes need to be considered separately. Moreover, most studies investigating the affinity ceiling have used haptens or simpler antigens, such as HEL, and do not consider complicated functional attributes of antibodies, such as neutralisation, breadth of recognition across variant antigens, or different target epitopes on a single antigen. Furthermore, most affinity and activation studies of more complex antigens, particularly HIV antigens, focus on affinity differences within a single epitope (often CD4bs) and therefore miss differences across epitopes^75,81,103,104^. Many HIV vaccine candidates, in particular those for germline targeting vaccines^23^, focus on VRC01-like bnAb development, where the goal is to increase germline precursors within the wider population and then use more traditional Env trimers to broaden the response^105,106^. However, VRC01-like bnAb development has yet to be achieved in a genetically diverse model following this strategy. Continued work in this area often focuses on continually increasing the affinity of candidate antigens for both germline and mature bnAbs beyond the affinity ceiling^25,103,107–109^. Our results indicate that this push towards continuously higher affinity antibodies may not be the optimal way to induce breadth or to promote bnAb expansion once the initial selection of the rare precursor pool has been initiated, particularly as a successful vaccine will likely need to induce multiple bnAbs. Our data also support the continuation of the established strategy of masking non-neutralising epitopes, given the high affinity of non-bNAbs and their comparable levels of B cell activation, and preferential germline activation. Particularly in light of recent evidence that suggests epitope masking by non-competitive non-bNAbs may work to enhance low-affinity B cell activation^110^. However, prior studies show that generally ‘redirecting’ the immune response to make bnAbs is not as simple as reducing the generation of non-bNAbs^111–116^. Further work is needed to understand how bnAbs develop and what precise cellular activation profiles are preferential for immunodominance to boost the induction of bnAbs. Such studies may require investigation of not only induction of B cell responses but also what impact activation has on downstream selection processes by the concurrent T cell response, and how that linkage may be best directed. Furthermore, these relationships between affinity activation should be further tested using membrane presentation of antigen to observe these interactions in a more physiologically relevant manner.

Overall, the data presented here suggest that rather than exclusively optimising candidate vaccine antigens for increasingly higher affinity for bnAbs and their precursors, there is a need for a more holistic approach. To build a more realistic picture of how an immune response might develop, investigation is needed of Env vaccine candidates that are best able to activate B cells bearing BCRs for bnAbs and their precursors and the consequences for downstream B cell selection and expansion. In particular, our results suggest that initial calcium flux is a much greater prediction of downstream processes, such as antigen internalisation, that are ultimately required for B cell selection, than in vitro measurements of mAb affinity.

## Methods

### Clinical sample ethics

The protocols and study documents for the clinical sampling were approved by the local Research Ethics Committee (REC) South Central - Hampshire B (REC 19/SC/0423, Vaccines in Chronic Infection study). All participants were recruited at the Mortimer Market Centre for Sexual Health and HIV Research (London, UK) or at the Ian Charleson Day Centre at the Royal Free Hospital (London, UK). Historical samples from people with detectable HIV viraemia or non-ART mediated viral control were collected according to study protocols approved by the local REC London-City & East (REC 12/LO/1572). All subjects enrolled in these studies provided written informed consent. The studies complied with all relevant ethical regulations for work with human participants and conformed to the Helsinki Declaration principles and Good Clinical Practice (GCP) guidelines.

### Antigen ELISA

Half-well 96-well MaxiSorp plates were coated with 100 μL per well of galvanathis lectin (GNL) diluted 1:50 in 0.1 M NaCO_3_ (SOSIP ELISA) or SARS-CoV-2 Spike protein (2ug/ml) at 4 °C overnight. Next day, plates were washed three times with PBS-T (PBS, 0.05% Tween) and blocked with assay buffer (PBS, 1% casein) for 1 h at room temperature. For SOSIP ELISA, plates were then incubated with AMC011, BG505 or B41 (also named 9032-08.A1.4685) SOSIPs at 2 μg/ml in assay buffer for 2 h at room temperature. Antibodies were diluted in assay buffer to 40 μg/ml, titrated 1:10, and 50 μl added to the plates. After 1 h incubation at room temperature, plates were washed three times with PBS-T before addition of 50 μL alkaline phosphatase-conjugated goat anti-human Fc IgG at 1:1000 in assay buffer and incubation for a further hour. Plates were washed six times with PBS-T before addition of 50 μL of colorimetric alkaline phosphatase substrate was added. Absorbance was measured at 405 nm.

### Biolayer interferometry

A Fortebio Octet Red instrument was used to assess binding kinetics, using immobilised IgG on streptavidin conjugated sensors bound with a biotinylated anti-human Fc IgG linker. Fresh sensors were used for each sample. Size-exclusion chromatography purified AMC011 or BG505 SOSIPs were used as free analyte in 50 mM Tris-HCl (pH 7.5). Biosensors (Sartorius) were hydrated in the relevant buffer for approximately 5 min prior to exposure to analyte. Baseline reading was obtained by immersing sensors in buffer for a further 5 min before loading with biotinylated anti-IgG linker at 5 μg/ml (ThermoFisher) for 2.5 min. Monoclonal antibodies were diluted to 2 μg/ml in buffer. Sensors were then immersed in target mAbs for 2.5 min before obtaining second baseline in buffer for 5 min. Association of SOSIP with immobilised IgG was measured over 30 min. SOSIP titrated first at 1:5 from 10x higher than published *K*_D_ values, then 1:2 for tighter affinity measurements. Subsequent disassociation was measured over 1 h. All experiments were conducted at 30 °C. Analysis was performed by ForteBio Discovery software using a 1:1 kinetic model, 1:2 models were also assessed to check for avidity interactions.

### Monoclonal antibody production

Filter sterile heavy and light chain Ig expression plasmids at 156 ng/ml were co-transfected using filter sterile PEImax (1 mg/ml) and Opti-MEM into 293 F expi cells in FreeStyle media at 1e6 cells/ml. Cells were cultured at 37 °C, 8% CO_2_. Supernatant was harvested post-transfection for IgG purification using Protein G beads. Briefly, supernatant was applied to the Protein G column at 4 °C overnight. Column was washed with PBS before antibodies were eluted with 0.1 M glycine (pH 2.2) into 2 M Tris-base (pH 9). Antibodies were then buffer exchanged into PBS using 50 KDa centrifugal concentrators.

### SOSIP protein production and purification

Filter sterile SOSIP and Furin expression plasmids at a 4:1 ratio were co-transfected using filter sterile PEImax (1 mg/ml) and Opti-MEM into 293 F expi cells in FreeStyle media at 0.5–0.7e6 cells/ml. Cells were cultured at 37 °C, 8% CO_2_. Supernatant was harvested seven days post-transfection, protease inhibitor was added to prevent degradation before purification via GNL-coated beads. Filtered supernatant was applied to the column at 4 °C overnight. The column was washed in 0.5 M NaCl, followed by 1X PBS, before elution in 1 M mannose, before buffer exchange into PBS.

### Mutant SOSIP production

Point mutations were created in the AMC011 SOSIP expression plasmid using the QuickChange Lightning site-directed mutagenesis (SDM) kit (Agilent) according to the manufacturer’s instructions. Following the SDM reaction, template plasmid was digested with DpnI, incubated for 30 min at 37 °C, followed by 20 min at 80 °C to inactivate DpnI. The resulting plasmids were transformed into DH5α competent E. coli cells (New England BioLabs). Mutations were confirmed using Plasmidsaurus whole plasmid sequencing. Mutations in BG505 SOSIP expression plasmid were performed by GenScript.

### Germline antibody production

ACS antibodies were reverted to their inferred germline sequence as previously described (Xiao et al.^79^; McGuire et al.^81^; Jardine et al.^21^). Briefly, VDJ sequences of mature antibodies were annotated using IMGT VQuest (Lefranc et al.,^117^), and non-CDR3 regions were reverted to their inferred allelic sequence. Computational methods are unable to fully deconvolute the germline CDR3 region, thus this was kept in the mature form (Jardine et al.^21^; Xiao et al.^79^). The same process was performed for the light chain VJ regions. The resulting inferred germline heavy and light chains were then cloned into antibody expression vectors and produced as monoclonal antibodies.

### TZM-bl luciferase neutralisation reporter assay

HIV-1 particles pseudotyped with BG505 or bal.26 Env proteins were produced in T75 flasks seeded the previous day with 3e6 cells HEK293T/17 cells in complete DMEM supplemented with 10% FBS, 100 IU/ml penicillin and 100 μg/ml streptomycin, grown at 37 °C, 5% CO_2_. Cells were co-transfected with HIV backbone plasmid PSG3delEnv using PEImax transfection reagent. Assays with AMC011 virus used full length AMC011 LAI virus, where cells were transfected with AMC011 LAI plasmid with PEImax. Supernatants were harvested 48 h post-transfection and stored at −80 °C. Neutralisation assays were conducted by serial dilution of mAbs in complete DMEM (10% FBS, 1% penicillin-streptomycin) and incubated for 1 h with pseudotyped virus at 37 °C. TZM-bl cells were added with 1:20 dilution of Dextran and incubated for 72 h at 37 °C. Luminescence was measured using the Bright-Glo luciferase kit (Promega) according to the manufacturer’s instructions. Measurements were performed in duplicate and used to measure 50% inhibitory dilution (ID50) values in GraphPad Prism software.

### Culture of Ramos 2G6 cells

All Ramos 2G6.4C10 (ATCC: CRL-1923) cells were grown in Iscove Modified Dulbecco’s Medium (IMDM, ThermoFisher) supplemented with 20% fetal bovine serum (FBS, ThermoFisher) and 5% penicillin/streptomycin (ThermoFisher), at 37 °C with 5% CO_2_. Cells were kept at densities between 0.2-1e6 cells/ml. For nucleofection, cells were transferred to IMDM, 20% FBS without penicillin/streptomycin for 24 h before and placed in antibiotic-free media for 24 h after, before being returned to 5% antibiotic media for subsequent culture.

### Nucleofection of B cells

Ramos B cells were cultured in IMDM media with 20% FCS and 100 IU/ml of penicillin/streptomycin, at 37 °C and 5% CO_2_. 24 h before engineering, cells were set to 0.2e6 cells per ml in antibiotic-free media. The following day 5e6 cells were used for engineering. Ribonucleoproteins (RNPs) were formed by combining sgRNAH296 (provided by Justin Taylor (Fred Hutchinson Cancer Research Center)) with *Sp*. Ca9 at a molar ratio of 2:1. Cells were combined with the RNP, then electroporated using the ThermoFisher Neon Electroporation kit according to the manufacturers instructions (voltage 1500, width 20, 1 pulse). Cells were rested for 30 min in antibiotic-free media before addition of AAV carrying the target mAb cassette at an MOI of 2.5 per 1e6 cells. After 24 h, cells were washed and cultured in IMDM (20% FCS, 100 IU/ml penicillin/streptomycin) for 5 days before FACS sorting for antigen-specific binding.

### AAV mAb production

Original AAV plasmid construct was provided by Justin Taylor (Fred Hutchinson Cancer Research Center, http://n2t.net/addgene:32395)^118^. Specific antibody cassette sequences were produced as linear DNA constructs by Genewiz. AAV plasmid construct was double digested using BbsI/ApaI enzymes and gel-purified to isolate digested plasmid construct. Linear DNA constructs were inserted using Gibson Assembly (New England Biolabs) according to the manufacturer’s instructions. Once each specific antibody AAV expression construct was purified, AAV was produced using HEK293T cells. The day before transfection, cells were seeded at 0.5 × 10^6^ cells/ml in T175 flasks. Cells were transfected with OptiMEM, PEI (10 mM, Sigma-Aldrich, branched), pDGM6 plasmid (Addgene, 110660) and AAV expression construct. Cells were cultured for 3 days, changing the media after the first 24 h. Virus was then harvested and purified by density gradient, titre was determined using a QuickTitre AAV Quantitation Kit (Cell BioLabs) according to the manufacturer’s instructions.

### Fluorescence activated cell sorting of engineered cells

One week after nucleofection, Ramos cells were stained with streptavidin-PE, streptavidin-APC, and Invitrogen Fixable Live/Dead Blue. Cells were then sorted on a BD FACSAria Fusion cell sorter. After sorting, cells were washed and transferred to 500 μL of IMDM media supplemented with 20% FCS and 100 IU/ml penicillin and 100 IU/ml streptomycin, and cultured at 37 °C, 5% CO_2_.

### Intracellular calcium flux

Cells were stained with 1 μM Fluo-4 (Invitrogen) in complete IMDM with 250 μM probenecid at 37 °C. Cells were then resuspended in 250 μM probenecid in complete IMDM and incubated again at 37 °C. Cells were then run on a BD Fortessa measuring AF488 MFI. Baseline MFI was recorded for 30 s before addition of the stimulant. Activation was then recorded for a further 2.5 min. Ionomycin (1 μg/ml) and anti-kappa/lambda (10 μg/ml) were used as positive controls. Antigen specific stimulation was determined using streptavidin tetramers of biotinylated AMC011 or BG505 SOSIPs, or SARS-CoV-2 Spike protein.

### Primary cell calcium flux

Primary B cells were magnetically isolated by negative selection from whole PBMCs using the MACS B cell isolation kit II according to the manufacturer’s instructions. Isolated B cells were then stained for memory phenotype in PBS on ice (CD19-BV786, CD27-BUV395, CD20-AF700, CD21-BV421, CD10-BV605, CD3-BV510, CD14-BV510, live-dead Aqua). After washing, cells were stained for intracellular calcium flux as before, with an additional 30 min rest period at 37 °C before being analysed. Cells were run at a high threshold flow rate to ensure a continuous stream of each B cell subset was recorded and to limit natural fluctuations in MFI. Baseline MFI was recorded for 20 s before addition of anti-kappa/lambda antibody. Activation was then recorded for a further 1.5 min.

### pHrodo-Red antigen-specific internalisation

Antigen tetramers were created by incubating 1 μg of streptavidin pHrodo-red (Thermo Fisher) per 6 μg of biotinylated BG505 or AMC011 SOSIP at room temperature. Ramos cells were plated at 0.5 million in IMDM (5% FCS, 100 U/ml penicillin/streptomycin) per well in a 96-well plate. Antigen tetramers were then added, and cells were incubated at 37 °C, 5% CO_2_. At baseline, 30 min, 1 h and 2 h, cells were transferred to a new 96-well plate containing 8% paraformaldehyde for fixation. Cells were analysed by flow cytometry on a BD Fortessa. Biotinylated anti-IgM (Jackson ImmunoResearch) antibody was used as a positive control (1 μg/ml).

### Statistical and bioinformatic analysis

All statistical analysis was carried out using GraphPad Prism 10.0. Where possible, data were determined to have a normal distribution according to Prism’s statistical normality tests. Thus, Welch’s *t*-tests were used with statistical significance set at *P* < 0.05. Statistical significance is indicated in each figure with an asterisk with relevant *P* values are indicated in figure legends or are listed in Supplementary table 1. Where appropriate, mean values across three experimental repeats are shown. Cliffs delta was applied to quantify the magnitude and direction of calcium flux trends across primary B cell subsets. Directional trends were inferred when the 95% confidence intervals did not include 0. For all Spearman’s rank correlations, statistical significance was set at *P* < 0.05. Spearman’s rank correlation coefficient (*r*) is indicated on the relevant graph. All protein structural analysis was performed using UCSF Chimera software. Structures were downloaded from RCSF PDB; relevant PDB codes are indicated in figure legends.

## Supplementary information

ReesSpear et al_Supp Figures_second review_resubmission

## Data Availability

All data generated or analysed during this study are included in this published article and its supplementary information files. AAV plasmids were derived from linearised AAV backbone (Addgene plasmid no. 32395; http://n2t.net/addgene:32395) and modified with the indicated published antibody sequences (accession numbers: KX610471 and KX610466 (ACS202); GU980702 and GU980703 (VRC01); JN201894 and JN201911 (PGT121); GU272045 and GU272046 (PG9); PX440408 - PX440411 (ACS212 and ACS242)). Further information and any unique plasmids and/or cell lines generated in the study are available from the corresponding author upon reasonable request.
