# Supplementary material for: High affinity is insufficient for strong B cell activation by HIV broadly neutralising antibodies
Source: NPJ Vaccines. 2026 May 2;11:138. doi: 10.1038/s41541-026-01468-y (PMC13342595; doi:10.1038/s41541-026-01468-y)
Supplement: Supplementary file 1 — ReesSpear et al_Supp Figures_second review_resubmission [file 41541_2026_1468_MOESM1_ESM.docx]

# Supplementary information for: High affinity is insufficient for strong B cell activation by broadly neutralising antibodies

*Chloe Rees-Spear^1^, Olivia Payne^1^, Emma Touizer^1^, Alan Kennedy^1^, Luke Muir^1^, Peter Thomas^1^,* *Alyssa Thomas De-Cruz^2^, Rachel A. McKendry^2^, Leo Swadling^1^, Marit Van Gils^2^, James Voss^3^, Laura E McCoy^1^**

[Supp. Fig. 1. Representative gating strategy of MACS isolated primary memory B cells 2](#_Toc192600840)

[Supp. Fig. 2 Representative flow plots of each bnAb cell line indicating efficiency of CRISPR editing 3](#_Toc192600841)

[Supp Fig 3. Neutralisation and BLI assays validate bnAb functionality 5](#_Toc192600842)

[Supp. Fig. 4 Binding kinetic values of indicated bnAbs against mutant AMC011 (ACS202) or BG505 (SOSIPs). 7](#_Toc192600843)

[Supp. Fig. 5 Validation of ACS autologous antibodies 8](#_Toc192600844)

[Supp. Fig. 6. Diagram of antigen internalisation assay 10](#_Toc192600845)

[Supp. Table 1 Statistical analysis of calcium flux AUCs 11](#_Toc192600846)

Supp. Fig. 1.

**Supp. Fig. 1. Representative gating strategy of MACS isolated primary memory B cells.** Gating on CD10- live cells to remove immature B cells before identification of CD19+ CD20+ B cells. Memory B cell populations determined by expression of CD27 and CD21 as activated memory (CD27+ CD21-), resting memory (CD27+ CD21+), naïve (CD27- CD21+) and atypical memory (CD27- CD21-).

Supp. Fig. 2


**Supp. Fig. 2 CRISPR editing of Ramos B cells. A,** Representative flow plots of each bnAb cell line indicating efficiency of CRISPR editing. Edited B cells identified by double positive binding to the inserted Strep-tag were subsequently sorted by FACS. B, Diagram of CRISPR induced class switching. Pre-complexed RNPs direct Cas9 to regions 5’ to IgM gene and 3’ to IgG3 gene, inducing natural class switching to IgG1. IgG+ cells are then sorted. C, Representative flow plots for each antibody after CRISPR-induced class switch. CRISPR editing ranges in efficiency between cell lines. Class switched cells were stained for IgM and IgG expression, and IgG+ cells were sorted by FACS. D, Quantification of BCR expression by MFI post-FACS. Cells were stained for IgM or IgG expression and MFI for 10,000 live cells were measured by flow cytometry (top). Cells were then stained for Strep-tag expression, and 10,000 live cells were further assessed by flow cytometry (bottom), expression of the edited BCR was measured as a percentage of total BCR expression. E, Raw (top) and normalised (bottom) calcium flux AUC for bnAb cell lines stimulated with AMC011 or BG505 SOSIP. Total AUC was calculated from an average of 3 repeats then normalised to MFI of stained BCR expression for each cell line. F, Percent Ag binding of all cell lines as measured by MFI of cells bound to fluorescently labelled BG505 or AMC011 SOSIP. Each SOSIP was tetramerised with streptavidin PE or APC, and positive cells were identified as double positive PE and APC. Diagram in B Created in BioRender. Rees-spear, C. (2025) https://BioRender.com/lh0xl1k.

Supp Fig 3.

**Supp Fig 3. Neutralisation and BLI assays validate bnAb functionality.** A, Neutralisation function of each bnAb was validated by Tzm-bl pseudovirus neutralisation assay against two Tier 2 viruses (AMC011 and BG505). IC50 values indicated by horizontal dashed line. B, Linear regression analysis of total affinity (K_D_) as measured by SPR or BLI and published neutralisation breadth of a range of HIV bnAbs against BG505, performed using Prism software. C, Validation of each bnAb affinity for AMC011 and BG505 SOSIP by BLI. Calculated line of best fit and resulting binding kinetics determined using Octet Discovery Software. D, Linear regression analysis of IC_50_ values and binding kinetics for ACS202, PGT121, VRC01, PG9 and PGT151 against AMC011 and BG505 SOSIP, performed using Prism software. E, IC50 values and kinetic binding measurements for indicated bnAbs against BG505 and AMC011 SOSIPs. IC50 values calculated from Tzm-bl pseudovirus neutralisation assay. Kinetic measurements made with BLI and calculated using Octet Discovery Software from curve of best fit. BnAbs used in correlation in B: PGV04 (Yasmeen et al., 2014), 10E8 (Irimia et al., 2017), VRC01 (Yasmeen et al., 2014), NIH45-46 (Schiffner et al., 2018), PGT151 (Blattner et al., 2014), PGDM1400 (Stiechen et al., 2016), 3BNC60 (Jardine et al., 2016), PGT128 (Blattner et al., 2014), 3BNC117 (Jardine et al., 2016), PGT145 (Stiechen et al., 2016), 3BC315 (Schiffner et al., 2018), 4E10 (Zhang et al., 2019), PGT121 (Schiffner et al., 2018), PG9 (Sanders et al., 2013), LN01 (Pinto et al., 2019), ACS202 (Van Gils et al., 2016), CH103 (Henderson et al., 2019), 2G12 (Sanders et al., 2013), PG16 (Sliepen et al., 2015), 8ANC195 (Schiffner et al., 2018), b12 (Purwar et al., 2018), 35O22 (Schiffner et al., 2018), PGT135 (Kong et al., 2013), PGT130 (Bruxelle et al., 2021), 4025 (Schiffner et al., 2018).

Supp. Fig. 4

**Supp Fig. 4. Binding kinetic values of indicated bnAbs against mutant AMC011 or BG505 SOSIPs.** Indicated point mutations were made individually in the SOSIP sequence by site-directed mutagenesis (QuickChange Lightning Kit, Agilent). Mutated SOSIP trimers were produced in HEK-293F expi cells and trimer purified by size-exclusion chromatography. Affinity was measured by BLI to confirm reduction in affinity at each bnAb epitope. AMC011 SOSIP was used for ACS202, BG505 SOSIP was used for PGT121, VRC01 and PG9. Kinetic values determined from line of best fit by Octet Discovery Software.

Supp. Fig. 5

**Supp. Fig. 5. Validation of ACS autologous antibodies.** A, ACS antibodies were validated for binding and neutralisation function by ELISA and Tzm-bl pseudovirus neutralisation assay (respectively) against two Tier 2 viruses, AMC011(autologous) and BG505 (heterologous), and Tier 1 virus bal.26. Neutralisation IC50 values are indicated with no neutralisation (>50ug/ul, grey), weak neutralisation (10-20ug/ul, green), intermediate neutralisation (1-10ug/ul, yellow) and strong neutralisation (>1ug/ul, red). Maximum percent neutralisation (MPN) is also indicated. Area under the curve (AUC) of binding in ELISA is normalised to blank values. B, Binding kinetic values of selected ACS antibodies against AMC011 and BG505 SOSIPs as measured by BLI. Values are calculated from line of best fit as determined by Octet Discovery Software. C, Representative flow data indicating success of CRISPR engineering of each ACS antibody into Ramos B cells. D, Area under the curve (AUC) and maximum percent neutralisation (MPN) for inferred germline ACS mAbs against autologous AMC011 SOSIP and virus, respectively. Affinity kinetic data for germline ACS202 and ACS212 against AMC011 SOSIP. E, ELISA binding curves (left) and pseudovirus neutralisation curves (right) for ACS202 and ACS212 mature and germline antibodies against AMC011 SOSIP and virus, respectively. SARS-CoV-2-specific CR3018 antibody is used as a negative control.

## Supp. Fig. 6.

**Supp. Fig 6. A, Diagram of antigen internalisation assay**. Biotinylated antigen is conjugated to pHrodo-strep before being added to B cells. As pHrodo progresses along the endocytic pathway it increases in fluorescence with increased endosomal acidity. Changes in fluorescence are measured by flow cytometry to create a time course of antigen internalisation. Diagram created in BioRender. Rees-spear, C. (2025) https://BioRender.com/meqktou.

## Supp. Table 1

|  | **IgM AMC011** | | | | **IgM BG505** | | | |
| --- | --- | --- | --- | --- | --- | --- | --- | --- |
|  | **PGT121** | **PG9** | **ACS202** | **ACS212** | **PGT121** | **PG9** | **ACS202** | **ACS212** |
| **VRC01** | **** | ** | **** |  | *** | ** | *** |  |
| **ACS202** | * | * |  |  | ns | *** |  |  |
| **PGT121** |  | ** |  |  |  | *** |  |  |
| **ACS212** |  |  | * |  |  |  | ns |  |
| **ACS242** |  |  | ** | **** |  |  | ** | *** |
|  | **IgG AMC011** | | | | **IgG BG505** | | | |
| **VRC01** | ** | * | *** |  | **** | ** | *** |  |
| **ACS202** | ns | * |  |  | **** | **** |  |  |
| **PGT121** |  | * |  |  |  | **** |  |  |
| **ACS212** |  |  | **** |  |  |  | *** |  |
| **ACS242** |  |  | **** | * |  |  | **** | ** |

Supp. Table 1. Statistical analysis of calcium flux AUC for Figs 3 and 5. Welch’s t-test. *P <0.05, **P<0.005, ***P<0.0005, ****P<0.0001
